# 

**Published:** 1933

**Authors:** 


### The Bristol Medico-Chirurgical Journal

A JOURNAL OF THE MEDICAL SCIENCES FOR THE WEST OF ENGLAND AND SOUTH WALES.

PUBLISHED UNDER THE AUSPICES OF

*THE BRISTOL MEDICO-CHIRURGICAL SOCIETY.*

EDITOR:

J. A. NIXON, C.M.G., M.D., F.R.C.P.

WITH WHOM ARE ASSOCIATED

E. W. HEY GROVES, M.S., F.R.C.S., B.Sc.
A. E. ILES, O.B.E., M.B., B.S., F.R.C.S.
A. RENDLE SHORT, B.Sc., M.D., B.S., F.R.C.S.

E. WATSON-WILLIAMS, M.C., Ch.M., F.R.C.S., Assistant Editor.

EDITORIAL SECRETARY:

A. L. FLEMMING, M.B., Ch.B.

---

"Scire est nescire, nisi id me

Scire alius sciret."

---

*VOL. L.*

BRISTOL: J. W. ARROWSMITH LTD.
London: J. W. Arrowsmith (London) Ltd.

R31
.B8

### Contents of Vol. L

| | Page |
|---|---|
| A Surgical Adventure: An Autobiographical Sketch. By ERNEST W. HEY GROVES, M.S., F.R.C.S. | 1 |
| Enlarged Prostate. By A. RENDLE SHORT, M.D., B.S., F.R.C.S. | 23 |
| Anterior Poliomyelitis, or Infantile Paralysis. By H. CHITTY, M.S., F.R.C.S. | 37 |
| Surgery for Pain. By ERNEST ROCK CARLING, F.R.C.S. | 81 |
| Thirty Years' Progress in the Study of Rheumatic Heart Disease. By the late CAREY F. COOMBS, M.D., F.R.C.P. | 93 |
| Familial Multiple Telangiectases of the Skin and Mucous Membranes. By GORDON R. SCARFF, M.B., CH.B., F.R.C.S. | 113 |
| The Need for a Standard Method of Estimating the Blood-Pressure. By G. ARBOUR STEPHENS, M.D. | 121 |
| The History of the Bristol Medico-Chirurgical Journal. By PATRICK WATSON-WILLIAMS, M.D. | 151 |
| Bristol's Contributions to Medical Progress | 165 |
| The Centenary of the Foundation of the Bristol Medical School | 183 |
| Schola Medicinæ (Review) | 199 |
| The Twenty-second Long Fox Memorial Lecture: Recent Advances in the Ætiology, Diagnosis and Treatment of Cancer. By CECIL A. JOLL, M.D., M.S., F.R.C.S. | 201 |
| The Present Position of Radium Therapy. By SYLVIA B. WIGODER, M.D., Ph.D., D.M.R.E. | 233 |
| Bacillus Coli Infections of the Female Urinary Tract. By H. J. DREW SMYTHE, M.D., M.S., F.R.C.S., F.C.O.G. | 243 |
| The Use of the Ventral Position in the Treatment of Tuberculous Disease of the Spine in Children. By K. H. PRIDIE, M.B., B.S., F.R.C.S. | 261 |
| Bristol University: Department of Preventive Medicine | 267 |
| REVIEWS OF BOOKS | 51, 127, 199, 271 |
| EDITORIAL NOTES | 57, 134, 149 |
| OBITUARIES | 59, 60, 62, 63, 135, 286, 287 |
| MEETINGS OF SOCIETIES | 65, 138, 293 |
| THE MEDICAL LIBRARY OF THE UNIVERSITY OF BRISTOL | 66, 142, 297 |
| PUBLICATIONS RECEIVED | 300 |
| LOCAL MEDICAL NOTES | 71, 147, 302 |

### The Medical Library of the University of Bristol,

WITH WHICH IS INCORPORATED

### The Library of the Bristol Medico-Chirurgical Society.

---

*The following donations have been received since the publication of the list in November,* 1932.

February, 1933.

| | | |
|---|---|---|
| Association of American Physicians (1) | .. | 1 volume. |
| W. S. Bainbridge, Esq. (2) | .. .. .. | 1 ,, |
| Brompton Hospital (3) | .. .. .. | 1 ,, |
| Carnegie Institution of Washington (4) | .. | 1 ,, |
| Dr. A. C. Fryer (5) | .. .. .. .. | 1 ,, |
| General Medical Council (6) | .. .. .. | 2 volumes. |
| V. B. Green-Armytage, Esq. (7) | .. .. | 1 volume. |
| Dr. G. Hadfield (8) | .. .. .. .. | 1 ,, |
| Imperial Cancer Research Fund (9) | .. .. | 1 ,, |
| Dr. A. Lumière (10) | .. .. .. .. | 2 volumes. |
| Dr. J. E. R. McDonagh (11) | .. .. .. | 1 volume. |
| J. E. MacKenty, Esq. (12) | .. .. .. | 1 ,, |
| Michell Clarke Fund (13) | .. .. .. | 46 volumes. |
| Dr. M. Nierenstein (14) | .. .. .. | 2 ,, |
| Rockefeller Foundation (15) | .. .. .. | 3 ,, |
| Smithsonian Institution (16) | .. .. .. | 2 ,, |
| Surgeon-General of the United States Army (17) | | 1 volume. |
| Université de Liége (18) | .. .. .. | 3 volumes. |

Unbound periodicals have also been received from Professor E. W. Hey Groves.

### THE ONE HUNDRED AND FORTY-THIRD LIST OF BOOKS.

The figures in round brackets refer to the figures after the names of the donors. The books to which no such figures are attached have either been bought from the Library Fund or received through the *Journal*.

**Achard, Ch.** .. .. *The Œdema of Bright's Disease*, translated by M. Marcus .. .. .. .. .. .. .. .. 1930

**Atkinson, E.M.** .. *Intracranial Suppuration* .. .. .. .. (13) 1932

**Bailey, H., and Love, R. J. M.** *A Shorter Practice of Surgery*. Vol. II. 1932

**Bainbridge, W. S.** .. *Report on Sixth International Congress of Military Medicine* .. .. .. .. .. .. .. 1931

**Ball, W. G., and Evans, G.** *Diseases of the Kidney* .. .. .. (13) 1932

**Bankart, A. S. B.** .. *Manipulative Surgery* .. .. .. .. (13) 1932

**Barger, G.** .. .. .. *Ergot and Ergotism* .. .. .. .. .. .. (13) 1931

**Beaumont, G. E., and Dodds, E. C.** *Recent Advances in Medicine* 6th Ed. (13) 1931

**Beneden, J. van** .. *Recherches sur l'Infection l'Hypersensibilité et l'Immunité* .. .. .. .. .. .. (18) 1932

*Bibliography of Hookworm Disease* .. .. .. .. .. .. .. .. (15) 1922

**Blacklock, D. B., and Southwell, T.** *A Guide to Human Parasitology* (13) 1931

**Boome, E. J., and Richardson, M. A.** *Nature and Treatment of Stammering* .. .. .. .. .. .. .. (13) [1931]

**Bray, G. W.** .. .. *Recent Advances in Allergy* .. .. .. (13) 1931

**Browning, E.** .. .. *The Vitamins* .. .. .. .. .. .. .. (13) 1931

**Buchanan** .. .. .. *Manual of Anatomy* .. 5th Ed., 2 vols. (13) 1931

**Cannon, A., and Hayes, E. D. T.** *The Principles and Practice of Psychiatry* .. .. .. .. .. .. .. (13) 1932

*Chinin Formeln* .. .. .. .. .. .. .. .. .. .. .. .. .. (14) —

**Choyce, C. C.** .. .. *System of Surgery* .. .. 3rd Ed., 3 vols. 1932

**Collie, J.** .. .. .. *Fraud in Medico-Legal Practice* .. .. (13) 1932

**Creed, R. S., and others.** *Reflex Activity of the Spinal Cord* .. (13) 1932

**Crowe, H. W.** .. .. *Handbook of the Vaccine Treatment of Chronic Rheumatic Diseases* .. .. .. 2nd Ed. 1932

**Culpin, M.** .. .. *Recent Advances in the Study of Psychoneuroses* (13) 1931

**Davenport, C. B.** .. *Genetical Factor in Endemic Goitre* .. (4) 1932

**Dixon, S.** .. .. .. *Relation of Food to Disease* .. .. .. (5) 1932

**Duke-Elder, W. S.** .. *Textbook of Ophthalmology*, Vol. I. .. (13) 1932

**Garot, L.** .. .. .. *Créatinurie et Carbonurie* .. .. .. (18) 1932

**Glaister, J.** .. .. *Textbook of Medical Jurisprudence and Toxicology* .. .. .. .. 5th Ed. (13) 1931

**Gray** .. .. .. .. *Anatomy* .. .. .. .. .. .. 25th Ed. (13) 1932

**Greig, D. M.** .. .. *Clinical Observations on the Surgical Pathology of Bone* .. .. .. .. .. .. .. .. .. (13) 1931

**Green-Armytage, V. B., and Datta, P. C.** *Textbook of Midwifery in the Tropics* .. .. .. .. .. .. .. .. (7) 1933

**Hadfield, G., and Garrod, L. P.** *Recent Advances in Pathology* .. (8) 1932

**Haldane, J. B. S.** .. *The Causes of Evolution* .. .. .. (13) 1932

**Harmer, W. D.** .. *The Relative Value of Radiotherapy in the Treatment of Cancers of the Upper Air Passages* .. .. .. .. .. .. .. .. (13) [1932]

**Hawk, P. B., and Bergeim, O.** *Practical Physiological Chemistry* 10th Ed. (13) 1931

**Herrick, C. J.** .. .. *An Introduction to Neurology* 5th Ed. (13) 1931

**Housden, L. G.** .. *Breast-fed Baby in General Practice* .. .. 1932

**Hutchison, R.** .. .. *Index of Treatment* .. .. .. .. ..10th Ed. 1931

*Imperial Cancer Research Fund. Tenth Scientific Report* .. .. (9) 1932

*Index Catalogue of the Library of the Surgeon-General's Office of the United States Army.* 3rd Series, Vol. 10 (17) 1932

**Johannessohn, F.** .. *Chinin in der Allgemeinpraxis* .. .. (14) 1930

**Kerley, P.** .. .. .. *Recent Advances in Radiology* .. .. (13) 1931

**Lapière, S.** .. .. *Le Mycosis Fongoïde* .. .. .. .. (18) 1932

**Lattes, L.** .. .. .. *Individuality of the Blood.* Translated by L. W. A. Bertie .. .. .. .. .. (13) 1932

**Lees, D.** .. .. .. *Diagnosis and Treatment of Venereal Disease.* 2nd Ed. (13) 1931

**Lewis, Thomas** .. .. *Clinical Electrocardiography* .. .. .. (13) 1931

**Lumière, A.** .. .. *Quelques travaux . . . de la tuberculose* (10) 1932

**Lumière, A.** .. .. *Tuberculose, Contagion, Hérédité,* Ed. 2 (10) 1931

**McCleary, G. F.** .. *National Health Insurance* .. .. .. .. 1932

**McDonagh, J. E. R.** *The Nature of Disease Journal.* Vol. I. (11) 1932

**McGregor, A. L.** .. *Synopsis of Surgical Anatomy* .. .. .. 1932

**McKenty, J. E.** .. *Infections of the Upper Respiratory Tract in the Etiology of Uveitis* .. .. .. (12) —

**Maingot, R. H.** .. *The Management of Abdominal Operations*.. 1931

**Maingot, R. H.** .. *The Injection Treatment of Varicose Veins* 1932

**Martindale and Westcott.** *The Extra Pharmacopoeia.* Vol. I. 20th Ed. 1932

**Maximow, A. A.** .. *A Textbook of Histology* .. .. .. (13) 1931

**Miller, E.** .. .. .. *Modern Psychotherapy* .. .. .. .. (13) [1930]

**Miller, G. S.** .. .. *Human Hair and Primate Patterning* .. .. 1931

**Minty, L. L. M.** .. *The Legal and Ethical Aspects of Medical Quackery* .. .. .. .. .. .. .. 1932

**Morgan, W. G.** .. *Functional Disorders of the Gastrointestinal Tract* .. .. .. .. .. .. .. .. (13) —

**Morton, H.** .. .. *Diseases of the Stomach* .. .. .. .. (13) 1931

**Muir, R., and Ritchie, J.** *Manual of Bacteriology* .. 9th Ed. (13) 1932

*Nouveau Traité de Medecine.* Vol. X. (in 2 vols.) .. .. .. (13) 1933

Perry, A., and Harvey, D. *General Nursing* .. .. .. .. .. 1932

Purves-Stewart, J. .. *The Diagnosis of Nervous Diseases.* 7th Ed. (13) 1931

*Report of the Mental Deficiency Committee.* 9 parts in 2 .. .. .. 1929

Rowntree, L. G., and Snell, A. M. *A Clinical Study of Addison's Disease* .. .. .. .. .. .. .. .. (13) 1931

Sicard, J. A., and Forestier, J. *The Use of Lipiodol in Diagnosis and Treatment* .. .. .. .. .. .. .. 1932

Sigerest, H. E. .. .. *Man and Medicine* .. .. .. .. .. (13) [1932]

Sinclair, M. .. .. *Fractures* .. .. .. .. .. .. .. (13) 1931

Spencer, H. A. .. *Lightning, Lightning-stroke and its Treatment* (13) 1932

Sutton, R. L. .. .. *Diseases of the Skin.* 2 Vols. 8th Ed. 1931

Ten Teachers .. .. *Midwifery* .. .. .. .. .. 9th Ed. (13) 1931

Tomson, W. B... .. *Fluid Milk* .. .. .. .. .. .. .. 1932

Turner, A. C. .. .. *Diseases of the E.N.T.* .. .. .. .. .. 1932

Villiger, E. .. .. *Brain and Spinal Cord.* 9th revised American edition (13) 1932

Walker, N... .. .. *Introduction to Dermatology* .. 9th Ed. 1932

Watkyn-Thomas, F. W., and Yates, A. L. *The Principles and Practice of Otology* .. .. .. .. .. .. .. .. 1932

Warren, S... .. .. *The Pathology of Diabetes Mellitus* .. (13) 1930

Wheeler and Jack .. *Handbook of Medicine* .. .. 9th Ed. (13) 1932

Woods, H. M., and Russell, W. *An Introduction to Medical Statistics* (13) 1931

### TRANSACTIONS, REPORTS, JOURNALS, ETC.

American Otological Society, Transactions. Vol. XXII. .. .. .. 1932

Association of American Physicians, Transactions. XLVII. .. (1) 1932

Brompton Hospital Reports. Vol. I. .. .. .. (3) Vol. I. 1933

Kelly. Directory of Bristol and Suburbs .. .. .. .. .. .. .. 1932

Medical Directory .. .. .. .. .. .. .. .. .. .. .. .. 1933

Medical Register. 2 Vols... .. .. .. .. .. .. (6) 1930, 1931

Methods and Problems of Medical Education. 21st Series .. (15) 1932

Report on the Mental Deficiency Committee [B.M.A.] .. .. .. 1932

Rockefeller Foundation, Annual Report .. .. .. .. .. (15) 1931

Smithsonian Institution, Annual Report, 1931 .. .. .. .. (16) 1932

Surgical Clinics of North America. Vol. 12, No. 5 .. .. .. .. 1932

### Publications Received

From Edward Arnold & Co.:

*A Doctor to a Mother.* By Eardley Holland, R. C. Jewsbury and W. Sheldon. Price 1s. 6d.

From Ash & Co. Ltd.:

*Influenza.* By T. B. Layton, D.S.O., M.S. (Lond.). Price 1s.

From John Bale, Sons and Danielson Ltd.:

*A Short History of Surgery.* By Sir D'Arcy Power, K.B.E., F.R.C.S. Price 3s. 6d.

From Cassell and Company, Ltd.:

*Injection Treatment in Medical Practice.* By David Levi, M.B., M.S. Price 6s.

From William Heinemann (Medical Books) Ltd.:

*Diseases of the Eye.* By A. Rugg-Gunn, M.B. Price 12s. 6d.

From H. K. Lewis & Co. Ltd.:

*Bacteriophage in the Treatment and Prevention of Cholera.* By J. Morison, C.I.E., M.B., D.P.H. Price 9s.

*Chronic Rheumatism and the Pre-rheumatic State.* By J. H. Hindley-Smith, M.A., M.R.C.S., L.R.C.P. Price 5s. 6d.

*Materia Medica for Nurses.* By A. Muir Crawford. 2nd Edition. Price 3s. 6d.

*The Early History of the Infant Welfare Movement.* By G. F. McCleary, M.D., D.P.H. Price 6s.

From Macmillan & Co. Ltd.:

*Diseases of the Heart.* By Sir Thomas Lewis, C.B.E., F.R.S. Price 12s. 6d.

From Oxford University Press:

*Human Values in Psychological Medicine.* By C. P. Blacker, M.C., M.A., M.D. Price 8s. 6d.

From Radiological Review Publishing Co.:

*Radiologic Maxims.* By Harold Swanberg, B.Sc., M.D. Price $1.50.

From John Wright & Sons Ltd.:

*The British Journal of Surgery.* January, 1933. Price 12s. 6d.

### Local Medical Notes

**Paul Bush Gold Medal.**—This medal has been awarded to Mr. A. A. G. Flemming, M.R.C.S., L.R.C.P.

APPOINTMENTS.

Brevet-Colonel H. E. Stanger-Leathes, I.M.S. (who was a resident officer at the Royal Infirmary in 1902), to be Honorary Surgeon to H.M. the King.

Professor V. B. Green-Armytage, I.M.S., to be Gynæcologist to the West London Hospital in succession to the late Sir Henry Simson.

J. E. Newton, M.B., Ch.B. (Bristol), to be non-resident Casualty Officer at the West London Hospital.

**Bristol Royal Infirmary.**—Consulting Physician: J. R. Charles, M.A., M.D., F.R.C.P.

**Bristol General Hospital.**—Consulting Physician to the Skin Department: W. Kenneth Wills, O.B.E., M.A., M.B., B.C. Physician to the Skin Department: Norman F. C. Burgess, M.D. Physician: Hugh H. Carleton, M.A., M.D., B.Ch. Assistant Physician: Charles B. Perry, M.B., Ch.B.

EXAMINATION RESULTS.

**University of London.**—M.D.—H. J. D. Smythe, M.S.

**University of Bristol.**—Students of the University have recently passed the following examinations:—

M.B., Ch.B. (*Part I.*).—*Second Examination. Pass: In Organic Chemistry*: E. M. Barber, Marjorie R. A Batten, A. N. Brimelow, Kathleen E. Byrt, Margaret H. Davies, Daphne V. Dennis, J. P. M. Forde, H. D. T. Gawn, M. E. M. Herford, F. J. W. Hooper, C. R. G. Howard, C. B. Jones, Jane Mackintosh, G. L. Page, A. N. H. Peach, P. B. Ryan, Margaret E. M. Slater, R. J. K. Tallack, M. W. N. Tribe, T. R. de V. Vallentin, Ernest Want.

M.B., Ch.B. (*Part II.*).—*Second Examination. Pass:* R. D. Cæsar, Monica L. Hawkins, J. S. W. Little (with distinction in Anatomy), N. R. Matheson, Coralie W. Rendle-Short, E. L. Ward.

B.D.S.—*Second Examination. Pass: In Dental Anatomy only:* C. A. Blanden.

L.D.S.—*First Professional Examination. Pass: In Dental Anatomy (Completing Examination):* B. E. ffrench, D. E. Royal. *In Anatomy and Physiology only:* R. E. Buchan. *In Dental Anatomy only:* J. G. Coates, R. A. Hilton, T. John, C. L. Read, A. J. Staple.

**Conjoint Board.**—M.R.C.S., L.R.C.P.—*Pass: In Physics and Elementary Biology:* P. W. J. Parkes. *In Elementary Biology:* M. Thompson. *In Materia Medica and Pharmacology:* F. L. Wollaston, G. M. Denning. *In Anatomy and Physiology:* G. W. Vowles. *In Medicine:* J. F. H. Eagles.* *In Pathology:* Edith K. Harris, Aldyth D. Jones, Violet Fry. *In Midwifery:* A. J. Board, S. B. Adams, Mary G. Thomas, G. L. Foss, R. H. Moodie, T. R. Aubrey, Gladys R. Llewelyn, A. V. House. *In Surgery:* E. O. Walker. *In Elementary Biology:* T. John.

L.D.S., R.C.S.—*First Professional Examination. Pass: Part I. (Dental Mechanics):* J. W. E. Snawdon, G. L. R. Welch. *Part III. (b) (Dental Anatomy-Physiology):* T. H. Williams.

L.D.S., R.C.S.—*Final Examination. Pass: Part I.:* B. H. W. Baker, A. C. Davies, D. Gent, N. G. Harrison, N. E. Miles, J. D. Rees, H. F. Vere. *Part II.:* Margaret D. Hooper.

* Qualified.

---

### National Health Insurance Acts Lectures.

### UNIVERSITY OF BRISTOL.

Arrangements have been made in conjunction with the British Medical Association for Dr. Jonas, of Barnstaple, to give two lectures on the National Health Insurance Acts on Thursdays, 27th April and 4th May, at 3 p.m., in the Anatomical Lecture Theatre of the University.

These lectures are designed primarily for final year medical students, but any medical practitioners who are interested are invited to attend.

---

As we go to press we learn with deep regret of the death of Dr. D. S. Davies. An Obituary notice will appear in the Summer issue.

### Bristol Medico-Chirurgical Society.

### President.

E. W. HEY GROVES, M.S., F.R.C.S., B.Sc. Lond.

### President-Elect.

H. ELWIN HARRIS, B.A., M.B. Cantab., F.R.C.S.

### Committee.

*Ex-Officio* J. A. NIXON, *C.M.G.*, B.A., M.D. Cantab., F.R.C.P. (Editor of *Journal*).

G. PARKER, M.A., M.D. Cantab. (Hon. Librarian).

*Elected.*

W. K. WILLS, *O.B.E.*, M.A., M.B., B.Ch. Cantab. (Ex-President).

DUNCAN WOOD, F.R.C.S.

H. CHITTY, M.S. Lond., F.R.C.S.

A. R. SHORT, B.Sc., M.D. Lond., F.R.C.S.

H. G. KYLE, M.A., M.D., B.Ch. Oxon.

W. P. KENNEDY, M.D. Dub.

W. A. JACKMAN, M.B., F.R.C.S. Ed.

H. ELWIN HARRIS, Jun., M.A., M.B. Cantab., F.R.C.S.

A. L. FLEMMING, M.B., Ch.B. Bristol.

### Honorary Secretary.

H. L. SHEPHERD, M.B., Ch.M. Brist.

### Honorary Treasurer.

A. E. ILES, *O.B.E.*, M.B., B.S. Lond., F.R.C.S.

### University Library Sub-Committee.

G. PARKER, M.A., M.D. Cantab.
A. R. SHORT, B.Sc., M.D., B.S. Lond., F.R.C.S.
J. O. SYMES, M.D. Lond.

Medical Practitioners wishing to join the Society are requested to communicate with the Hon. Secretary, Mr. H. L. SHEPHERD, 23 Pembroke Road, Clifton. The Annual Subscription is £1 11s. 6d., payable to Hon. Treasurer.

---

*Extracts from Journal By-laws.*

4.—The *Journal* shall be delivered free to Subscribers and also to Members of the Society whose subscriptions are not in arrear.

6.—The Annual Subscription to the *Journal* is 10/6, payable to Hon. Treasurer.

---

Communications intended for insertion in the *Journal* should be sent to the Editor, Dr. J. A. NIXON, 7 Lansdown Place, Clifton, Bristol.

Books for Review and Exchange Journals should be sent to the Assistant-Editor, *Bristol Medico-Chirurgical Journal*, Medical Library, The University, Bristol.

Communications referring to the delivery of the *Journal* should be sent to the Editorial Secretary, A. L. Flemming, 48 Pembroke Road, Clifton, Bristol.

Cases for Binding Vols. I—XLIX are now ready, and may be obtained price 1/9 each (postage extra), upon application to J. W. ARROWSMITH LTD., Quay Street, Bristol; or the *Journal* will be bound, including Case, for 7/- per volume.

### Bristol Medico-Chirurgical Society.

### PAST PRESIDENTS.

1874—75. FREDERICK BRITTAN, M.D., B.S. Dub.

1875—76. ROBERT WILLIAM COE.

1876—77. SAMUEL MARTYN, M.D. Ed.

1877—78. AUGUSTIN PRICHARD, M.D. Berlin.

1878—79. HENRY EDWARD FRIPP, M.D. Würzburg.

1879—80. WILLIAM MICHELL CLARKE.

1880—81. JOSEPH GRIFFITHS SWAYNE, M.D. Lond.

1881—82. EDWARD LONG FOX, M.D. Oxon.

1882—83. JAMES GEORGE DAVEY, M.D. St. And.

1883—84. WILLIAM JOHNSTONE FYFFE, M.D., B.A. Dub.

1884—85. GEORGE FORSTER BURDER, M.D. Aberd.

1885—86. WILLIAM HENRY SPENCER, M.D., M.A. Cantab.

1886—87. FRANCIS POOLE LANSDOWN.

1887—88. LEMUEL MATTHEWS GRIFFITHS.

1888—89. ROBERT SHINGLETON SMITH, M.D., B.Sc. Lond.

1889—90. NELSON CONGREVE DOBSON, Ch.M. Brist.

1890—91. SAMUEL HENRY SWAYNE.

1891—92. FRANCIS RICHARDSON CROSS, M.B. Lond., LL.D. Brist.

1892—93. EDWARD MARKHAM SKERRITT, M.D., B.S., B.A. Lond.

1893—94. JAMES GREIG SMITH, M.B., C.M., M.A. Aberd., F.R.S.E.

1894—95. ALFRED JAMES HARRISON, M.B. Lond.

1895—96. ARTHUR WILLIAM PRICHARD.

1896—97. ALFRED EDWARD AUST LAWRENCE, M.D., C.M. Aberd.

1897—98. JOHN EDWARD SHAW, M.B., C.M. Ed.

1898—99. ROBERT ROXBURGH, M.B. Ed.

1899—00. WILLIAM HENRY HARSANT.

1900—01. DAVID SAMUEL DAVIES, M.D. Lond.

1901—02. BARCLAY JOSIAH BARON, M.B., C.M. Ed.

1902—03. GEORGE MUNRO SMITH, M.D. Brist.

1903—04. JAMES PAUL BUSH, C.M.G., C.B.E., Ch.M. Brist.

1904—05. REGINALD EAGER, M.D. Lond.

1905—06. JOHN DACRE.

1906—07. JAMES TAYLOR.

1907—08. HENRY WALDO, M.D., C.M. Aberd.

1908—09. JOHN MICHELL CLARKE, M.D., M.A. Cantab.

1909—10. JAMES SWAIN, C.B., C.B.E., M.D., M.S. Lond.

1910—11. BERTRAM MITFORD HERON ROGERS, M.D., B.Ch., B.A. Oxon.

1911—12. CHARLES ALEXANDER MORTON.

1912—13. WALTER CARLESS SWAYNE, M.D., B.S. Lond. and Brist.

1913—14. PATRICK WATSON-WILLIAMS, M.D. Lond.

1914—15. WILLIAM HARRY CHRISTOPHER NEWNHAM, M.A., M.B. Cantab.

1915—16. GEORGE PARKER, M.A., M.D. Cantab.

1916—17. FRANCIS HENRY EDGEWORTH, M.A., M.D. Cantab., D.Sc. Lond.

1917—18. FREDERICK LACE.

1918—19. ROBERT GUTHRIE POOLE LANSDOWN, M.D., B.S. Durh.

1919—20. I. WALKER HALL, M.D., Ch.B. Vict.

1920—21. LEOPOLD ERNEST VALENTINE EVERY-CLAYTON, M.D. Lond.

1921—22. CYRIL HUTCHINSON WALKER, B.A., M.B. Cantab.

1922—23. JOHN ALEXANDER NIXON, C.M.G., B.A., M.D. Cantab.

1923—24. JOHN LACY FIRTH, M.D., M.S. Lond.

1924—25. JOHN ODERY SYMES, M.D. Lond.

1925—26. THOMAS CARWARDINE, M.S. Lond.

1926—27. ARTHUR LAUNCELOT FLEMMING, M.B., Ch.B. Brist.

1927—28. WALTER KENNETH WILLS, M.A., M.B., B.Ch. Cantab.

1928—29. HENRY LAWRENCE ORMEROD, M.D., B.Ch. R.U.I.

1929—30. CHARLES FERRIER WALTERS.

1930—31. DAVID CHARLES RAYNER, Ch.M. Brist.

1931—32. JOHN ROGER CHARLES, M.A., M.D. Cantab.

### 1933.

### LIST OF SUBSCRIBERS

TO THE

### Bristol Medico-Chirurgical Journal.

### HONORARY MEMBERS OF THE SOCIETY.

*Swain, James, *C.B.*, *C.B.E.*, M.D., M.S. Lond. .. .. .. .. Wyndham House, Easton-in-Gordano.

*Watson-Williams, P., M.D. Lond. 2 Rodney Place, Clifton, Bristol 8.

*Smith, W. A., M.A., M.B. Oxon. 100 Glebe Road, Cambridge.

### SUBSCRIBERS.

*Those marked * are Members of the Society.*

*Ackland, W. R., M.R.C.S., L.D.S. ... ... ... 5 Rodney Place, Clifton, Bristol 8.

*Adams, A. W., M.S. Lond. ... ... ... ... ... Rodney House, Clifton, Bristol 8.

*Aldwinckle, H. M., M.B., Ch.B. Brist. ... 1 Manor Road, Fishponds, Bristol.

*Alexander, D. A., M.B., Ch.B. Vict. ... ... 112 Pembroke Road, Clifton, Bristol 8.

Alexander, G. L., B.A., M.B., B.Ch. Cantab. West African Medical Service, Laura, via Accra, Gold Coast.

*Alford, A. F., M.B., Ch.B. Brist. ... ... ... 14 Clifton Park, Bristol 8.

Alford, H. J., M.D. Lond. ... ... ... ... ... 4 Park Terrace, Taunton.

*Armstrong, Jean, M.B., Ch.B. Brist. ... ... Succoth, Duckmoor Road, Ashton Gate, Bristol 3.

Astley-Weston, B.A., M.B., Ch.B. Brist. ... High Meadow, Radbrook, Shrewsbury.

*Aubrey, T., M.B. Lond. ... ... ... ... ... Bitton, near Bristol.

*Baker, Lily A., M.D., B.Ch., B.A.O., R.U.I. Clifton Down House, Beaufort Buildings, Clifton, Bristol 8.

*Barber, M. C., M.B., Ch.B. Brist. ... ... ... Ingledene, High Street, Staple Hill, Bristol.

*Barker, G. H., M.D., B.Sc. Lond. ... ... ... 124 Redland Road, Bristol 6.

*Bartholomew, E. U. ... ... ... ... ... ... ... 12 Lansdown Place, Clifton, Bristol 8.

*Bates, R. M. ... ... ... ... ... ... ... ... ... Purdown House, Stapleton, Bristol 5.

Beatson, D. H., M.B., Ch.B. Brist. ... ... ... 8 Richmond Park Road, Clifton, Bristol 8.

*Bergin, F. G. ... ... ... ... ... ... ... ... 31 Oakfield Road, Clifton, Bristol 8.

Bernard, C. ... ... ... ... ... ... ... ... ... 564 Fishponds Road, Fishponds, Bristol.

Berry, F. C., M.D., B.Ch., B.A. Dub. ... ... Locarno, Burnham, Somerset.

*Berry, R. J. A., Prof., M.D., C.M. ... ... ... Director of Medical Services, Stoke Park Colony, Bristol 5.

*Birrell, J. A., M.D., M.S. Lond. ... ... ... 36 Kellaway Avenue, Bristol 6.

*Blachford, J. V., *C.B.E.*, M.D. Durh. ... ... Milverton House, Long Ashton, near Bristol.

Blackett, J. F., M.D., B.S. Lond. ... ... ... Hamsterley, Bloomfield Park, Bath.

Bletchly, G. Playne, M.B. Lond. ... ... ... Hazelwood, Nailsworth, Glos.

*Bodman, A. P., M.B., Ch.B. ... ... ... ... 11 Hurle Crescent, Clifton, Bristol 8.

*Bodman, C. O., M.D. Durh. ... ... ... ... 17 Upper Belgrave Road, Clifton, Bristol 8.

*Bodman, F. H., M.B., Ch.B. Brist. ... ... ... 2 Redland Court Road, Bristol 6.

*Bodman, J. Hervey, M.D. Lond. ... ... ... 11 Hurle Crescent, Clifton, Bristol 8.

*Boulton, B. J., M.B., Ch.B. Brist. ... ... ... Ham Green Hospital, Pill, near Bristol.

*Bown, N. J., M.B., Ch.B. Brist. ... ... ... ... Demeter, Wraxall, Somerset.

*Bradbeer, E. G., M.B., Ch.B. Brist. ... ... Hillside, Cranbrook Road, Bristol 6.

*Bradley, W. H., B.A., D.M. Oxon. ... ... St. Wulstan's, Stratton-on-Fosse, Bath.

| | |
|---|---|
| *Brierley, C. E ... ... ... ... ... ... ... ... | Great Totton, Hants. |
| *Brinckman, W. P., M.B., Ch.B. Brist. ... ... | 4 Oakland Road, Redland, Bristol 6. |
| *Brittain, H. A., M.B., Ch.B. Dub. ... ... ... | "Kilronan," Anglesea Road, Dublin, Ireland. |
| *Britton, R. B., M.B., B.S. Lond. ... ... ... | 33a Whiteladies Road, Clifton, Bristol 8. |
| *Brocklehurst, R. J., M.A., D.M. Oxon.... ... | The University, Bristol 8. |
| *Buckmaster, G. A., M.A., M.D. Oxon. ... ... | 6 Victoria Square, Clifton, Bristol 8. |
| *Burges, R. ... ... ... ... ... ... ... ... ... | Druid's Mead, Stoke Bishop, Bristol 9. |
| *Burgess, N. F. C., M.D. Cantab.... ... ... ... | Hereford House, Clifton, Bristol 8. |
| *Burnett, R. H., M.B., B.S. Durh. ... ... ... | 479 Bath Road, Brislington, Bristol 4. |
| *Bush, G. B., M.B., Ch.B. Brist. ... ... ... | 5 Lansdown Place, Clifton, Bristol 8. |
| *Cairns, F. J. ... ... ... ... ... ... ... ... ... | Devon House, Whitehall Road, Bristol 5. |
| *Carey, R. S., *O.B.E.*, B.A. Cantab. ... ... | 502 Bath Road, Brislington, Bristol 4. |
| *Carleton, H. H., M.A., M.D., B.Ch. Oxon. ... | 1 Lansdown Road, Clifton, Bristol 8. |
| *Carwardine, Thomas, M.B., M.S. Lond.... ... | 16 Victoria Square, Clifton, Bristol 8. |
| *Casson, E., M.D., Ch.B. Brist. ... ... ... ... | Dorset House, Clifton Down, Bristol 8. |
| *Cates, H. J., M.D. Lond. ... ... ... ... ... | Northwoods House, Winterbourne, Bristol. |
| *Cave, Edward J., M.D. Lond. ... ... ... ... | 16 The Circus, Bath. |
| *Chambers, E. R. ... ... ... ... ... ... ... ... | 9 Clifton Park, Clifton, Bristol 8. |
| *Charles, J. R., M.A., M.D. Cantab.... ... ... | 5 Rodney Place, Clifton, Bristol 8. |
| *Chesterman, J. T., M.D., B.S. ... ... ... ... | Glen Avon, Broomfield Road, Bath. |
| *Chitty, H., M.S. Lond. ... ... ... ... ... | 46 Pembroke Road, Clifton, Bristol 8. |
| *Christofferson, P. E., M.B., Ch.B. Brist. ... | 8 Lansdown Place, Clifton, Bristol 8. |
| *Clarke, R. C., *O.B.E.*, M.B., Ch.B. Brist. ... | 29 Victoria Square, Clifton, Bristol 8. |
| *Coates, Vincent M., *M.C.*, M.A., M.D., B.C. Cantab. ... ... ... ... ... ... ... ... | 10 The Circus, Bath. |
| Connellan, P. S. ... ... ... ... ... ... ... ... | Marmion, Shortheath Road, Farnham, Surrey. |
| *Cookson, R. G. ... ... ... ... ... ... ... ... | 5 Worcester Road, Clifton, Bristol 8. |
| *Coombs, N. C., M.R.C.S., L.R.C.P. ... ... ... | 3 Pembroke Road, Clifton, Bristol 8. |
| *Cooper, William Russell ... ... ... ... ... ... | 13 Westfield Park, Redland, Bristol 6. |
| *Corfield, C., M.D. Durh. ... ... ... ... ... ... | 5 Kensington Place, Clifton, Bristol 8. |
| *Cory, R. A. S.; M.B., Ch.B. Brist. ... ... ... | Montego Bay, Jamaica, B.W.I. |
| *Cossham, W. L., M.B., Ch.B. Brist. ... ... | 3 Claremont Road, Bishopston, Bristol 7. |
| *Courtney, R. M., M.B., Ch.B. Glasg. ... ... | 487 Gloucester Road, Bristol 7. |
| *Coxon, Beatrice .. ... ... ... ... ... ... ... | 16 Richmond Hill, Clifton, Bristol 8. |
| Cridland, A. B. ... ... ... ... ... ... ... ... | Salisbury House, Chapel Ash, Wolver-[hampton. |
| *Crook, B. A., M.B., Ch.B.Brist. ... ... ... | Timsbury. |
| *Crossman, F. W., M.B. Durh. ... ... ... ... | White's Hill, Hambrook, near Bristol. |
| *Collinson, E. M. D. M., M.B., Ch.B. Brist. ... | 15 All Saints Road, Clifton, Bristol 8. |
| *Cross, Clara D., M.D. ... ... ... ... ... | 17 Midford Road, Odd Down, Bath. |
| *Dacre, John ... ... ... ... ... ... ... ... ... | 14 Eaton Crescent, Clifton, Bristol 8. |
| *Datta, S. S. M.D. Brist. ... ... ... ... ... ... | Snowdon House, Fishponds, Bristol. |
| *Davies, E. D. D. ... ... ... ... ... ... ... ... | Standish House, Stonehouse, Glos. |
| *Dawson, W. R., *O.B.E.*, M.D. Dub.... ... ... | 18 Brock Street, Bath. |
| Devine, H., *O.B.E.*, M.D. Lond. ... ... ... | Holloway Sanatorium, Virginia Water. |
| *Dix, C. ... ... ... ... ... ... ... ... ... ... | 11 Victoria Square, Clifton, Bristol 8. |
| *Dixon, Helen M., M.B., Ch.B. Brist. ... ... | 10 Whatley Road, Clifton, Bristol 8. |
| *Dixon, T. B. ... ... ... ... ... ... ... ... ... | 11 Pembroke Road, Clifton, Bristol 8. |
| *Duerden, J. R., M.B., Ch.B. Brist. ... ... | 149 Bell Hill Road, St. George, Bristol 5. |
| *Dutton, Hilda R. ... ... ... ... ... ... ... | 405 Stapleton Road, Bristol 5. |
| *Dyer, Walter Percy ... ... ... ... ... ... ... | West View, Westbury-on-Trym, Bristol. |
| *Eglinton, J. H. C. ... ... ... ... ... ... ... | Street, Somerset. |
| Elliot Bros. Ltd. ... ... ... ... ... ... ... | 20–22 O'Connell Street, Sydney, N.S.W., Australia. |
| Elliott, F. Percy, M.B., C.M. Aberd. ... ... | 113 Grove Rd., Walthamstow, London, E.17. |
| *Elliott, W. H. A., M.B., B.S. Lond. ... ... | 116 Pembroke Road, Clifton, Bristol 8. |

| | |
|---|---|
| *Fallon, Col. J. ... | 35 Henleaze Gardens, Henleaze, Bristol. |
| *Faraker, E. C. ... | 85 Coldharbour Road, Westbury Park, Bristol 6. |
| Farnfield, W. W. ... | Clive Vale, Gillingham, Dorset. |
| *Farquharson, J. L. ... | Breda, Weston-super-Mare. |
| *Fawn, G. F. ... | 6 Oakfield Road, Clifton, Bristol 8. |
| *Fayle, B. W. D., M.B., Ch.B. ... | Reine Barnes, Dursley, Glos. |
| *Fells, A., M.B., C.M. Edin. ... | 6 Elton Road, Clifton, Bristol 8. |
| *Fells, G. R., M.B., Ch.B. Brist. ... | Eaton House, Southmead Road, Bristol. |
| *Fells, R. R., M.B., B.S. Lond. ... | 12*d* Cotham Road, Bristol 6. |
| *Feneley, G. L., M.B., Ch.B. ... | Englewood, Stoke Lane, Westbury-on-Trym. |
| *Firth, J. L., M.D., M.S. Lond. ... | 8 Victoria Square, Clifton, Bristol 8. |
| *Fisher, A. C., M.B., Ch.B. Brist. ... | Kalene Hill, Mwini Lunga, N.W. Rhodesia. |
| *FitzGibbon, G. M., M.D. ... | The General Hospital, Bristol 1. |
| *Flemming, A. A. G. ... | The Royal Infirmary, Bristol 1. |
| *Flemming, A. L., M.B., Ch.B. Brist. ... | 48 Pembroke Road, Clifton, Bristol 8. |
| *Flemming, C. E. S. ... | Manvers House, Bradford-on-Avon. |
| *Forrester-Brown, Maud, M.D., M.S. Lond. ... | 22 Coombe Park, Bath. |
| *Foss, E. V., M.D., Ch.B. Brist. ... | Summer Hill House, St. George, Bristol 5. |
| *Fox, F. E., B.A. Camb. ... | Brislington House, Brislington, Bristol 4. |
| *Fraser, A. D., M.A., M.B., Ch.B. Glasg. ... | 4 Alexandra Road, Clifton, Bristol 8. |
| *Fraser, D., M.B., Ch.B. Edin. ... | Westgate, Dursley, Glos. |
| *Freeth, H., M.D. Dub. ... | 93 Redland Road, Bristol 6. |
| *Fuller, H. L. ... | 9 Gay Street, Bath. |
| *Garden, R. R., M.A., M.B., B.Ch. Aberd. ... | 9 Harley Place, Clifton, Bristol 8. |
| Garland, E. B., M.B., C.M. Edin. ... | 114*a* Hampton Road, Redland, Bristol 6. |
| *Gee, C. A. H., M.B., Ch.B. Brist. ... | Portbury, Nr. Bristol. |
| *Glenny, E. T., M.B., B.S. Lond. ... | 102 Pembroke Road, Clifton, Bristol 8. |
| *Gordon, R. G., M.D., B.Sc. Edin. ... | 9 The Circus, Bath. |
| *Gorham, A. P., M.B., Ch.B. Brist. ... | 53 Westbury Road, Westbury-on-Trym. |
| *Gornall, W. A., M.D., Brist. ... | Orchard House, Nailsea, Somerset. |
| *Greenlaw, Madge E., M.B., Ch.B. Brist. ... | 194 Redland Road, Bristol 6. |
| Griffiths, Cornelius A. ... | 35 Newport Road, Cardiff. |
| *Groves, E. W. Hey, M.D., M.S. Lond. ... | 25 Victoria Square, Clifton, Bristol 8. |
| *Hall, E. George, M.B. Lond. ... | 42 Apsley Road, Clifton, Bristol 8. |
| *Hall, I. Walker, M.D., Ch.B. Vict. ... | Shortwood Lodge, Pucklechurch, Glos. |
| *Ham, C. I., M.B., Ch.B. Brist. ... | 16 Elm Lane, Redland, Bristol 6. |
| *Harris, H. Elwin, B.A., M.B. Cantab. ... | 13 Lansdown Place, Clifton, Bristol 8. |
| *Harris, H. E., M.A., M.B., B.Ch. Cantab. ... | 13 Lansdown Place, Clifton, Bristol 8. |
| *Harrison, L. P. (Miss), M.B., Ch.B. ... | Homœopathic Hospital, Bristol 8. |
| *Hartley, G., M.B., B.S Lond. ... | 28 Pembroke Road, Clifton, Bristol 8. |
| *Hay, C. P., M.B., Ch.B.Edin. ... | 30 Cairns Road, Westbury Park, Bristol 6. |
| *Hector, F., M.D. Lond. ... | 11 Clifton Hill, Clifton, Bristol 8. |
| *Henderson, James, M.B., Ch.B. Aberd. ... | The City Sanatorium, Yardley Road, Birmingham. |
| *Herapath, C. E. K., *M.C.*, M.D. Lond. ... | Ormlie, Clifton, Bristol 8. |
| *Heron, Gordon, M.B., Ch.B. Brist. ... | 34 Gloucester Road, Bristol 7. |
| *Heron, Gordon, Mrs., M.B., Ch.B. Brist. ... | 34 Gloucester Road, Bristol 7. |
| *Hooker, J. A., M.B., Ch.B. Brist. ... | 16 St. Edyth's Road, Sea Mills 9. |
| *Hosken, J. G. F. ... | Uley, Glos. |
| Hospital, Bristol General (*Secretary*, T. W. Gregg). | |
| *Houghton, Lydia M., M.B., B.S. Lond. ... | 74 Church Road, Redfield, Bristol 5. |
| *Hughes F. W. Terrell, M.B., Ch.B. Brist. ... | Chew Magna, Somerset. |
| *Hunter, F. S. ... | c/o Messrs. J. Wright & Sons Ltd., Publishers, Bristol 1. |

| | |
|---|---|
| *Iles, A. E., *O.B.E.*, M.B., B.S. Lond. | 17 Victoria Square, Clifton, Bristol 1. |
| Infirmary, Bristol Royal (*Secretary*, E. C. Smith). | |
| Ingle, C. Durban | Somerton, Somerset. |
| *Irving, J. T., M.A., M.D., Ph.D. | Department of Physiology, The University, Bristol 8. |
| *Jackman, W. A., M.B., Ch.B. Brist. | 15 Mortimer Road, Clifton. Bristol 8. |
| *James, J. A., M.D. Lond. | 5 Rodney Place, Clifton, Bristol 8. |
| *James, April D., M.B., Ch.B. Brist. | The Eye Hospital, Lower Maudlin Street, Bristol. |
| *Jenkins, R. D. | Almondsbury, nr. Bristol. |
| *Jenkins, F. G., M.B., Ch.B. Brist. | 51 Redcliff Hill, Bristol 1. |
| *Johnson, R. G., M.B. Lond. | 119 Redland Road, Bristol 6. |
| *Joll, C. A., M.D., M.S. Lond. | 64 Harley Street, London, W.1. |
| *Jordan, L. R., M.B., Ch.B. Bristol. | Southmead Hospital, Bristol. |
| *Joscelyne, P. C., M.B., Ch.B. Brist. | 70 Longmead Avenue, Bishopston, Bristol 7. |
| *Kemm, N. F., M.B., B.S. Lond. | 188 Redland Road, Bristol 6. |
| *Kennedy, W. P., M.D. Dub. | 3 Gay Street, Bath. |
| *Kerfoot, Stanley J. | 194 Redland Road, Bristol 6. |
| *Kingston, S. H., M.B., Ch.B. Brist. | 3 Whiteladies Road, Clifton, Bristol 8. |
| *Kyle, H. G., M.A., M.D., B.Ch. Oxon. | 31 Westbury Road, Bristol. |
| *Lace, F. | 5 Gay Street, Bath. |
| *Langley, G. F., M.B., Ch.B. Brist. | The Manse, Trentham Road, Lougton, Stoke-on-Trent. |
| *Lees, E. Leonard, M.D., C.M. Edin. | 22 Richmond Hill, Clifton, Bristol 8. |
| *Legat, R. Eddowes, M.B., C.M. Edin. | Murray House, Staple Hill, Bristol. |
| Leigh, W. W. | Llansamnor House, Cowbridge, Glam. |
| *Leonard, R. C., M.D. Durh. | Bower Ashton, Bristol. |
| *Levis, J. S., *M.C.*, M.B., B.Ch., N.U.I. | 20 Gay Street, Bath. |
| *Linton, Marion S., M.B. Lond. | 21 Oakfield Road, Clifton, Bristol 8. |
| *Lister, A. E. J., M.B., B.S. Lond. | 86 Pembroke Road, Clifton, Bristol 8. |
| *Lister, L. Margaret | 12 All Saints Road, Clifton, Bristol 8. |
| *Logan, H. B. | Elmwood, Aberdeen Road, Cotham, Bristol 6. |
| *Lucas, R. P., M.B., Ch.B. Brist. | 15 Edgecumbe Road, Redland, Bristol 6. |
| *Lucas, J. E. | 48 Coronation Road, Bristol 3. |
| *Lucas, J. J. S., M.D. Lond. | 186 Wells Road, Totterdown, Bristol 4. |
| *Lysaght, A. C. | 8 Clifton Hill, Bristol, Bristol 8. |
| *Macfie, J. D., M.B., Ch.B. Glasg. | Winsley Sanatorium, near Bath. |
| Mackinnon, Charles, M.B., C.M., M.A. Glasg. | Davaar, Cirencester. |
| *MacLachlan, A. M., M.B., Ch.B., Glasg. | 65 Redcliff Hill, Bristol 1. |
| Maclean, Sir Ewen J., M.D., C.M. Edin. | 12 Park Place, Cardiff. |
| *MacLeod, Donald, M.B., C.M. Edin. | 84 Redland Road, Redland, Bristol 6. |
| *MacWatters, J. C., *M.B.E.* | Almondsbury, near Bristol. |
| *Marshall, A. H., M.B., Ch.B. Bristol. | Odelos, Canford Lane, Bristol. |
| Marshall, A. L., M.B., B.A. Cantab. | Raveningham, Norwich. |
| *Martin, P. S. | Victoria Quadrant, Weston-super-Mare. |
| Mather, J. S., M.B., C.M. Aberd. | Lawrence Hill House, Bristol 5. |
| *Mayes, F. J. A. | 79 Pembroke Road, Clifton, Bristol 8. |
| *McCrea, Phillip, M.D., B.Ch. Dub. | 56 Kellaway Avenue, Bristol 6. |
| *McDowell, A., M.B., Ch.B. Edin. | The General Hospital, Bristol 1. |
| *McKenzie, W. G., *M.C.* | Ministry of Health Regional Med. Office, 3 Victoria Road, Darlington, Co. Durham |
| *McLannahan, J. G. | The Mount, Stonehouse. |
| *Meadows, G. | W. D. & H. O. Wills, No. 1 Factory, East Street, Bedminster, Bristol 3. |
| Miall, C. L'O. | Elm Hayes, Paulton, near Bristol. |
| *Milling, T., M.B., Ch.B. Belfast | 1 Vicarage Road, Southville, Bristol 3. |
| *Milner, A., M.B., B.Ch. Dub. | 61 Cotham Brow, Bristol 6. |

| | |
|---|---|
| *Moore, R. M., M.A., M.B., Ch.B. Cantab. ... | Mundens, Malmesbury, Wilts. |
| *Moore, C. A., M.B., M.S. Lond. ... ... ... | 56 St. Paul's Road, Clifton, Bristol 8. |
| *Morris, L. N., M.B., Ch.B. Brist. ... ... ... | 24 All Hallows Rd., Upper Easton, Bristol 5. |
| *Mundy, G. S., M.B., B.Ch. Brist. ... ... ... | Homewood, Coombe Dingle, Bristol. |
| *Murphy, F. D., M.B., Ch.B., B.Sc. Cork ... | Queen Mary's Hospital, Stratford, London, E. |
| *Myles, G. T. ... ... ... ... ... ... ... ... ... | 7 Apsley Road, Clifton, Bristol 8. |
| *Myles, Q. St. L., M.A., B.M., B.Ch. Oxon. | 7 Apsley Road, Clifton, Bristol 8. |
| *Neild, Newman, M.B., B.Ch. Vict. ... ... ... | 9 Richmond Hill, Clifton, Bristol 8. |
| *Newnham, W. H. C., M.B., M.A. Cantab. ... | 3 Lansdown Place, Victoria Square, Clifton, Bristol 8. |
| *Nixon, J. A., *C.M.G.*, M.D. Cantab. ... ... | 7 Lansdown Place, Clifton, Bristol 8. |
| *Noble, J. Innes, M.B., Ch.B. Liverp. ... ... | 102 Pembroke Road, Clifton, Bristol 8. |
| *Nott, Winifred, M.B., Ch.B. Brist.... ... ... | "Fassiefern," Upper Cranbrook Road, Redland, Bristol 6. |
| *O'Brien, C., M.B., Ch.B. ... ... ... ... ... | The Children's Hospital, Bristol 6. |
| *O'Donohoe, Monica A., M.B., Ch.B. ... ... | 13 Blenheim Road, Redland, Bristol 6. |
| *Ormerod, G. L., M.A., M.B., B.Ch. Cantab. ... | 23 Southfield Road, Westbury-on-Trym, Bristol. |
| Orr-Ewing, H. J., *M.C.*, M.D. Lond. ... ... | English Mission Hospital, Jerusalem. |
| *Parker, George, M.A., M.D. Cantab. ... ... | 9 Pembroke Road, Clifton, Bristol 8. |
| *Parkes, J. A., M.B., Ch.B. Liverp. ... ... ... | 8 South Road, Redland, Bristol 6. |
| Parkinson, Lt.-Col. G. S., *D.S.O.*, R.A.M.C. ... | R.A.M.C. Mess, Grosvenor Place, London, S.W.1. |
| *Parry, R. H., M.B., B.S. Lond. ... ... ... | Newby, Holmes Grove Road, Henleaze. |
| Parsons, Sir J. H., M.B., B.S., B.Sc. Lond. ... | 54 Queen Anne Street, London, W. |
| *Paul, R. G. ... ... ... ... ... ... ... ... | The Royal Infirmary, Bristol 1. |
| *Pearce, J. L., M.B., Ch.B. Edin. ... ... ... | Hambrook Court, near Bristol. |
| *Perry, Bruce, M.B., Ch.B. Brist. ... ... ... | 4 Richmond Park Road, Clifton, Bristol 8. |
| *Phillips, P., M.B., Ch.B. Brist.... ... ... ... | Southmead Hospital, Bristol. |
| *Pinkerton, J. M., B.Sc. Wales; M.B., Ch.B. Glasg. | 5 The Circus, Bath. |
| *Pollard, J., M.B., B.Ch., B.A.O., N.U.I. ... | 88 Coronation Road, Bristol 3. |
| *Potter, Mabel F., M.B., Ch.B. ... ... ... | 1 Mortimer Road, Clifton, Bristol 8. |
| *Powell, H. F., M.D. Brux. ... ... ... ... | Ellenborough Park, Weston-super-Mare. |
| *Powell, J. J., M.D. Lond. ... ... ... ... | 2 Gloucester Road, Bishopston, Bristol 7. |
| *Price, A., M.B., Ch.B. Brist. ... ... ... ... | 16 Cavendish Road, Henleaze, Bristol. |
| *Price, N. L., M.B., Ch.B. Brist. ... ... ... | 16 Vicarage Road, Southville, Bristol 3. |
| *Pridie, K. H., M.B., B.S. Lond.... ... ... ... | Frenchay Orthopædic Hospital, Bristol. |
| *Pringle, Miss J. L., M.B., Ch.B. (Edin.) ... | 115 Lower Oldfield Park, Bath. |
| *Prowse, D. C., M.B., Ch.B. Brist. ... ... ... | Wigmore House, Thornbury, nr. Bristol. |
| *Pyke, H. D., M.B., Ch.B.Brist.... ... ... ... | 88 Redland Road, Bristol 6. |
| *Rankin, P. C., M.B., Ch.B.Glasg. .. .. .. | Lawrence Hill House, Bristol. |
| *Rayner, D. C., Ch.M. Brist. ... ... ... ... | 9 Lansdown Place, Clifton, Bristol 8. |
| *Redman, Ethel, M.B., Ch.B. ... ... ... ... | Winford Orthopædic Hospital, nr. Bristol. |
| *Robertson, D., M.B., Ch.B. Edin. ... ... ... | 205 Wells Road, Knowle, Bristol 4. |
| *Rogers, Herbert, M.B., Ch.B. Brist. ... ... | 11 Clifton Hill, Bristol 8. |
| *Rolfe, R., B.A., M.B., B.C. Cantab. ... ... | Park House, St. Andrew's Road, Avonmouth. |
| *Rowley, A. T. ... ... ... ... ... ... ... | Wrington. |
| *Rudolf, G. R. A. de M. ... ... ... ... ... ... | Brentry Colony, Westbury-on-Trym, Bristol. |
| Savage, W. G., M.D., B.Sc. Lond. ... ... ... | "Bourn," 7 Trewartha Park, Weston-super-Mare. |
| *Saxby, Ida, D.Sc., Lond. ... ... ... ... ... | 24 Durdham Park, Bristol 6. |
| *Scarff, G., M.B., Ch.B. Edin. ... ... ... ... | 5 Rodney Place, Clifton, Bristol 8. |
| *Scott, H. H. ... ... ... ... ... ... ... ... | 78 Old Market Street, Bristol 1. |
| *Shaw, J. E., M.B., C.M. Edin. ... ... ... ... | 23 Caledonia Place, Clifton, Bristol 8. |

| | |
|---|---|
| *Shepherd, H. L., M.B., Ch.M. Brist. | 23 Pembroke Road, Clifton, Bristol 8. |
| *Short, A. R., B.Sc., M.D., B.S. Lond. | 69 Pembroke Road, Clifton, Bristol 8. |
| *Smith, G. Cockburn, M.D. Brux. | 12 South Road, Newton Abbot. |
| *Smith, T. Wilson, M.D. Lond. | 26 The Circus, Bath. |
| *Smythe, H. J. D., *M.C.*, M.D., M.S. Lond. | 15 Eaton Crescent, Clifton, Bristol 8. |
| *Somers, Mary F. E. | 2 Ashcombe Road, Weston-super-Mare. |
| *Spoor, A., M.B., B.Ch. Cantab. | The Hydro, Bristol 1. |
| *Staniland, M. F. | 255 Stapleton Road, Bristol 5. |
| *Statham, R. S. S., *O.B.E.*, M.D., M.Ch. Brist. | 23 Pembroke Road, Clifton, Bristol 8. |
| *Steven, G. D., M.B., Ch.B.Edin., D.P.H. | 11 North Parade, Bath. |
| *Stone, D. M., M.D., B.S. Lond. | 28 Pembroke Road, Clifton, Bristol 8. |
| *Strover, H. W M., *O.B.E.*, M.B., Ch.B. Aberd. | 24 Gloucester Road, Bishopston, Bristol 7. |
| *Sutton, G. E. F., M.D. Lond. | 1 Pembroke Road, Clifton, Bristol 8. |
| *Symes, J. O., M.D. Lond. | 6 Pembroke Vale, Clifton, Bristol 8. |
| | |
| *Tasker, D. G. C., M.B., M.S. Lond. | 9 College Fields, Clifton, Bristol 8. |
| *Taylor, A. L., M.D. Lond. | The General Hospital, Bristol 1. |
| Taylor, A. L. | The Royal Infirmary, Bristol 1. |
| *Taylor, H. N., M.A. Cantab., M.D. Dub. | Hillside, Ebbw Vale. |
| *Temple, G. H., M.B., C.M. Edin. | Ailanthus, Weston-super-Mare. |
| Thin, James | 54 & 55 South Bridge, Edinburgh. |
| *Thomas, J. D., M.B. Cantab. | Dan-y-Graig, Cheyne Road, Stoke Bishop, Bristol 9. |
| *Thom du Toit, G., M.R.C.S., L.R.C.P. | Bristol Royal Infirmary. |
| *Thompson, J., M.B., Ch.B. Edin. | Eastbourne House, Devizes. |
| *Todd, A. T., *O.B.E.*, M.B., Ch.B. Brist. | 10 Tyndall's Park Road, Clifton, Bristol 8. |
| *Trist, J. R. R., *M.C.* | 120 Henleaze Road, Henleaze. |
| *Trueman, R. S., B.Sc., L.R.C.P., M.R.C.S. | Bristol Royal Infirmary. |
| *Tryon, Victoria S., M.B., Ch.B. Brist. | 3 Harley Place, Clifton, Bristol 8. |
| | |
| *Walbank, A., M.B., Ch.B. Leeds | 30 Whiteladies Road, Clifton, Bristol 8. |
| *Walker, Cyril H., B.A., M.B. Cantab. | 8 Oakfield Road, Clifton, Bristol 8. |
| Wallace, S., O.B.E. | 10 Knightstone Rd., Weston-super-Mare. |
| *Walsh, M. J., M.B., Ch.B. Dub. | 2 Knowle Road, Bristol 4. |
| *Walters, C. F. | 5 Mortimer Road, Clifton, Bristol 8. |
| *Wansbrough, T. B., M.B., Ch.B. Brist. | 8 Mortimer Road, Clifton, Bristol 8. |
| *Wanklyn-James, C. W., *O.B.E.* | 3 Mortimer Road, Clifton, Bristol 8. |
| *Warren, R., M.A., M.D. Oxon. | Ellenborough Hall, Weston-super-Mare. |
| *Waterhouse, R., M.D. Lond. | 3 The Circus, Bath. |
| *Watson-Williams, E., *M.C.*, B.A., M.B., Ch.M. Cantab. | 12 Victoria Square, Clifton, Bristol 8. |
| *Weddell, C. W. | Boydville, 46 Miller St., West Melbourne Australia. |
| Weston, B. A., M.B. (see Astley-Weston). | |
| *Whelton, A., B.A., M.B., Ch.B. Cork | Innisfree, Western Road, Cork. |
| *White, E. B. C. | Mental Hospital, Fishponds, Bristol. |
| Whitehouse, H. Beckwith, M.S. Lond. | 62 Hagley Road, Edgbaston, Birmingham. |
| *Wigoder, Sylvia Beatrice, M.A., M.D., Ph.D. | Radium Centre Royal Infirmary, Bristol 1. |
| Willcox, R. Lewis | 97 London Road, Salisbury. |
| Williams, S. R., M.B. Lond. | Buckfastleigh, Devon. |
| *Williams, A. R., M.B., Ch.B. Brist. | 1 Horsley Hill Road, South Shields. |
| *Wills, W. K., *O.B.E.*, M.B., B.C. Cantab. | 1 Victoria Square, Clifton, Bristol 8. |
| *Wood Duncan | 5 Eaton Crescent, Clifton, Bristol 8. |
| *Wood, W. V., *M.C.*, B.A. Cantab. | Henley Lodge, Yatton, Som. |
| *Wright, A. J. M.B., B.S. Lond. | Clifton Park, Clifton, Bristol 8. |
| | |
| *Zealand, L., M.D. Man. | Brecon Lodge, Westbury-on-Trym, Bristol. |

